# Actinic cheilitis in rural workers: prevalence and associated factors

**DOI:** 10.31744/einstein_journal/2022AO6862

**Published:** 2022-05-18

**Authors:** Maria Helaynne Diniz Faria, Luanna Mayrany Alves Costa Silva, Rodrigo Porpino Mafra, Marquiony Marques dos Santos, Samara Carollyne Mafra Soares, Jamile Marinho Bezerra de Oliveira Moura

**Affiliations:** 1 Universidade do Estado do Rio Grande do Norte Caicó RN Brazil Universidade do Estado do Rio Grande do Norte, Caicó, RN, Brazil.; 2 Universidade Federal do Rio Grande do Norte Natal RN Brazil Universidade Federal do Rio Grande do Norte, Natal, RN, Brazil.

**Keywords:** Cheilitis, Rural population, Ultraviolet rays, Prevalence, Pathology, oral

## Abstract

**Objective:**

To evaluate the prevalence of actinic cheilitis in rural workers and factors associated with the development of this condition.

**Methods:**

A cross-sectional study was conducted in a city in Northeastern Brazil. Data were collected by clinical examination and use of a questionnaire validated with 300 rural workers. The χ^2^ test was employed to identify possible associations between the presence of actinic cheilitis and clinical and demographic variables. Multiple logistic regression analysis was performed using forward stepwise selection. A p value of 0.05 was considered significant.

**Results:**

The prevalence of actinic cheilitis was 12.0% in the sample. The highest prevalence of actinic cheilitis was observed in white males, with low educational level, and an approximately 40-year history of sun exposure. Chronic lesions were commonly found in the lower lip and were characterized by scaling, dryness, and mild edema. Skin color, sex, educational level of patients, and cumulative sun exposure (in years), were identified as predictors of development of actinic cheilitis.

**Conclusion:**

Our results suggest the need to implement educational health strategies aimed to orient the population about risk factors and preventive measures of the disease. Appropriate clinical management of patients with actinic cheilitis is important for prevention of lip cancer.

## INTRODUCTION

Actinic cheilitis (AC) is a potentially malignant lesion affecting the lips, described as a degenerative condition of the lining epithelium, caused by the cumulative effect of solar ultraviolet (UV) radiation. The lower lip is more often affected because of the greater exposure of this anatomical site to solar radiation.^( 1 )^ It is estimated that almost 95% of cases of squamous cell carcinoma of the lip are preceded by AC, indicating a public health problem.^( 2 )^

The main etiological factor associated with AC is chronic exposure to solar UV radiation, especially type B (UVB), which has a greater potential to penetrate cells.^( 1 )^ The frequency of sun exposure without appropriate protection, the intensity of solar radiation, and the degree of skin pigmentation also influence in the development of this lesion.^( 3 )^ The risk of malignant transformation of AC may even be higher in the presence of other factors, such as smoking and alcohol drinking.^( 4 )^

The global prevalence of AC ranges from 15.5% to 43.2%.^( 1 , 5 )^ This disease is commonly identified in white men aged over 40 years, with history of chronic sun exposure.^( 6 , 7 )^ There are two clinical forms of AC: acute and chronic. The acute form is characterized by mild erythema, fissures, ulcerations and crusts, and less common, and results from short-term excessive sun exposure.^( 6 , 8 )^ Spontaneous resolution of these clinical alterations is frequently observed. On the other hand, the chronic form is caused by prolonged and cumulative exposure to UV radiation and is characterized by dry lips, fissures, discrete and diffuse swelling, loss of the border between the lip mucosa and skin, and leukoplastic plaques. Chronic AC occurs mainly in the fifth decade of life.^( 9 , 10 )^

According to the international literature, Brazil is the country with the highest levels of damaging UV radiation,^( 1 )^ a fact that favors the development of malignant lesions, such as lip cancer.^( 2 )^ Additionally, a semiarid climate predominates in Northeastern Brazil, with high levels of solar radiation and average monthly sunshine of 250 hours.^( 1 )^ Furthermore, outdoor occupations, such as fishing, mining and agriculture predominate in the region, which represent risk activities. In view of the chronic exposure to solar radiation and the lack of photoprotective measures, such as the appropriate use of sunscreens and hats, these workers are more likely to develop AC lesions and tend to be diagnosed late, which could be partly explained by the absence of symptoms.^( 1 , 9 , 11 )^

A recent study involving 10 oral pathology centers in different regions of Brazil analyzed cases of AC seen between 1953 and 2018, and demonstrated an increased incidence of this condition over the past decades.^( 2 )^ This finding justifies further research on the epidemiological and clinical characteristics of AC, whose early diagnosis and treatment are important because of its malignant potential.

## OBJECTIVE

To evaluate the prevalence of actinic cheilitis in rural workers and factors associated with the development of this condition.

## METHODS

### Study design and location

This was a cross-sectional study using an observational, exploratory, descriptive, and analytical approach. The study was conducted in the municipality of Caicó, RN, Brazil, located inland, in the western Seridó microregion, which corresponds to the 4^th^ Regional Public Health Pole. The estimated population is 68,343 inhabitants and the human development index is low (0.710). According to data from the National Meteorology Institute *(Instituto Nacional de Meteorologia)* a semiarid climate predominates in Caicó, RN. The average temperature ranges from 36°C to 40.3°C and the average monthly sunshine was 229.2 hours, in 2019.

### Study population and sample

The target population of this study was a group of trade union rural workers from the municipality of Caicó, RN. The sample size was calculated assuming a prevalence of AC of 43.24%,^( 11 )^ for a finite population of 5,000 rural workers of both sexes. The estimated number of participants was 294. Adding 10% for losses, the final sample was 323 rural workers. A total of 23 individuals refused to participate in the study. Thus, the sample consisted of 300 participants.

The following inclusion criteria were adopted: being duly registered in the Union of Rural Workers and Family Rural Farmers (STTR - *Sindicato dos Trabalhadores Rurais e Agricultores Rurais Familiares* ) of Caicó, RN; performing the profession for more than 14 years, the estimated period necessary to observe a possible cumulative effect of solar radiation and to develop AC;^( 9 )^ signing the free informed consent form.

### Training

Before data collection, two researchers underwent interexaminer reliability calibration process, divided into two steps. The first step consisted of discussion of AC according to the classification of Miranda et al.,^( 9 )^ and Poitevin et al.,^( 12 )^ including the study of clinical images. The second step comprised the calibration itself, when the researchers individually analyzed 30 images containing lip alterations of AC, with varying degree of severity. The interexaminer Kappa coefficient (κ) was 0.90, a value considered satisfactory for data collection.

### Data collection

The data were collected between November 2019 and March 2020 at the STTR of Caicó, RN, and at rural workers associations linked to STTR, where the participants were informed about the objective and benefits of the study. For data collection, an individual and structured questionnaire was employed, which was adapted from the questionnaire validated by Lucena et al.^( 1 )^ This questionnaire addressed sociodemographic variables (sex, age, skin color, and individual monthly income), occupational information, and health data (cumulative sun exposure in years, daily and weekly sun exposure, use of photoprotective measures, habits such as smoking and alcohol drinking). Smoking was classified based on the number of cigarettes smoked in the last 30 days. Alcohol drinking was considered in cases of consumption of two or more standard doses per day following the definition of a standard dose of the Brazilian Ministry of Health. Additionally, the use of health services and frequency of visits to the dentist were analyzed, as well as the participants’ level of knowledge on AC.

After employing the questionnaire, two researchers examined the lip mucosa of the participants following the clinical classification proposed by Miranda et al.,^( 9 )^ and Poitevin et al.,^( 12 )^under room light, using disposable latex gloves and surgical masks. The lesions were classified according to location (lower or upper lip), clinical presentation (acute or chronic), and degree of severity (mild, moderate, or severe).

The presence of scaling, dryness, and mild edema was used for the definition of mild cases of AC. Moderate cases were defined in the presence of erythema, fissures, red and/or white areas, and more marked edema and scaling. For the definition of severe cases, in addition to the features of moderate AC, the presence of ulcerations and crusts, hardened areas, more marked white and red areas, and atrophy were considered.^( 9 , 12 )^ Photographs of the lesions were obtained with the camera of a Xiaomi Mi A2^®^ mobile phone (Xiaomi Tech, China).

Patients diagnosed with AC were referred to the Stomatology service of the dental clinics of the *Universidade do Estado do Rio Grande do Norte* (UERN), in Caicó, RN, Brazil.

### Statistical analysis

The data were analyzed using descriptive and inferential statistics. Quantitative variables, such as age, duration of sun exposure, and level of tobacco and alcohol beverage consumption, were categorized based on a higher risk of AC, while the other dependent variables were categorized based on the median. Pearson’s χ^2^ test was applied to identify associations between AC and clinical and demographic variables.

Factors associated with AC were identified by multiple logistic regression analysis using a forward stepwise selection procedure. The p value to enter the model was set at <0.20. The software SPSS 25.0 (SPSS Inc., Chicago, IL) was used for all analyses considering a level of significance of 5% (p≤0.05).

### Ethical considerations

This study was conducted according to the ethical guidelines of Resolution 466/2012 of the National Health Council, and also the Declaration of Helsinki (2000), and was approved by the Research Ethics Committee (Approval # 3.101.702, CAAE: 03410918.7.0000.5294).

## RESULTS

Among the 300 rural workers examined, 189 (63.0%) were male and 111 (37.0%) were female. The mean age was 54 (SD±10.7) years. Regarding educational level, most participants (n=184; 61.3%) had incomplete elementary school, while 62 (20.7%) were illiterate and 39 (13.0%) had completed elementary school. Only 15 (5.0%) participants reported having completed high school. The mean individual monthly income (in *Reals* , R$) was R$ 916,05 (SD±546.49), equivalent to US$ 170.03 (SD±101.43).

There was a higher frequency of brown-skinned workers (n=178; 59.7%), followed by white (n=107; 35.6%) and black (n=15; 5.0%). The mean cumulative sun exposure in years was 41.7 (SD±11.9). Regarding working hours, 250 (83.3%) reported working two shifts (morning and afternoon). An expressive proportion of the sample (n=275; 91.6%) reported using some type of photoprotection against solar radiation, such as hat (n=132; 44.0%), cap (n=57; 19.0%), lip sunscreen (n=4; 1.3%), or more than one of these options (n=82; 27.3%).

**Figure 1:**
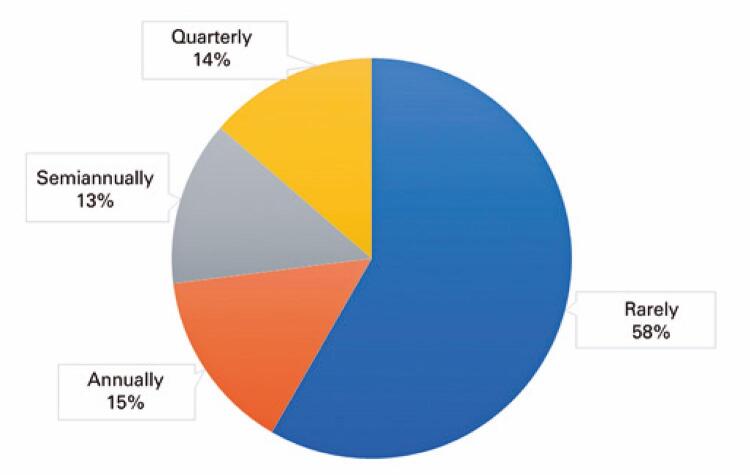
Frequency of rural workers seeking dental treatment

With respect to life and health habits, tobacco and alcohol consumption was reported by 105 (35.0%) participants, especially by male workers. Regarding access to health services, 297 (99.0%) reported having sought some type of service, with medical and dental services being the most cited (n=177; 59.0%). Figure 1 shows the frequency of rural workers seeking dental treatment.

**Figure 2:**
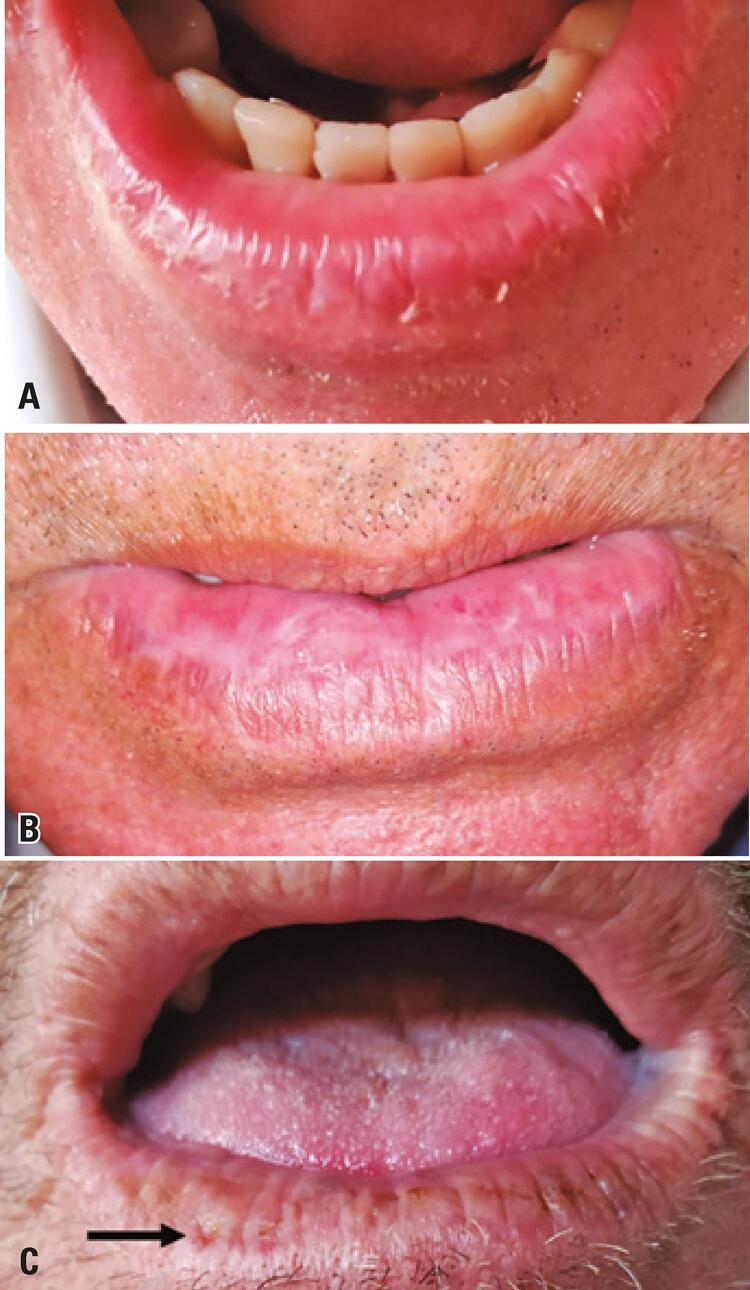
Clinical features observed in actinic cheilitis. A) Mild actinic cheilitis characterized by scaling, dryness, and mild edema in the lower lip; B) Moderate actinic cheilitis showing fissures, areas of leukoplasia and erythema, and more pronounced edema and scaling; C) Severe actinic cheilitis. Note the presence of atrophy, fissures, crusts, and pale and/or brownish spots in the lower lip

The prevalence of AC was 12% in the sample studied. Among the 36 cases of AC, 30 participants (83.4%) had chronic AC and 6 (16.6%) had acute AC. All cases were identified in the region of the lower lip. Regarding the severity of AC, all acute cases were classified as mild. Among the cases of chronic AC, 20 (66.6%) were mild, nine (30.0%) were moderate, and only one case (3.3%) was severe. The most common clinical findings in chronic AC lesions are illustrated in figure 2 . Only 13 (4.33%) rural workers reported being aware of AC, particularly its etiology.

Thirty (83.3%) participants diagnosed with AC were men and six were women. The most affected age range was 52 to 84 years. Bivariate analysis showed a significant association between the presence of AC and male sex (p=0.002), non-use of lip balm with sun protection factor (SPF) (p=0.001), and use of cocoa butter (p=0.025). On the other hand, smoking or alcohol consumption were not significantly associated with the presence of AC ( Table 1 ).

**Table 1:** Sociodemographic and occupational variables and habits associated with actinic cheilitis in rural workers

Variables	Presence of actinic cheilitis		PR	95%CI	p value^#^
	Yes (%)	No (%)			
Sex					
Male	31 (16.4)	158 (83.6)	3.64	1.46-9.09	0.002^†^
Female	5 (4.5)	106 (95.5)			
Age (years)					
23-51	10 (9.5)	95 (90.5)	1.00		0.260
52-58	17 (16.2)	88 (83.8)	0.59	0.28-1.22	
59-84	9 (10.0)	81 (90.0)	0.95	0.40-2.24	
Educational level					
Illiterate	6 (9.7)	56 (90.3)	1.00		0.130
Elementary school	28 (14.6)	164 (85.4)	0.66	0.29-1.53	
High school	2 (4.3)	44 (95.7)	2.23	0.47-10.53	
Income in R$*					
Up to 1.045,00	12 (12.4)	85 (87.6)	1.05	0.55-2.00	0.891
>1.045,00	24 (11.8)	179 (88.2)			
Working hours					
Two shifts	33 (13.2)	217 (86.8)	2.20	0.70-6.89	0.153
One shift	3 (6.0)	47 (94.0)			
Photoprotection					
No	0 (0.0)	25 (100.0)	1.15	1.10-1.20	0.054
Yes	36 (13.1)	239 (86.9)			
Use of sunscreen					
No	29 (12.0)	213 (88.0)	0.94	0.44-2.04	0.879
Yes	7 (12.7)	48 (87.3)			
Use of cap/hat					
No	8 (8.0)	92 (92.0)	0.56	0.27-1.19	0.121
Yes	28 (14.2)	169 (85.8)			
Use of lipstick					
No	36 (12.8)	246 (87.2)	0.87	0.83-0.91	0.230
Yes	0 (0.0)	15 (100.0)			
Use of lip balm with SPF					
No	32 (11.0)	260 (89.0)	0.14	0.08-0.24	0.001^†^
Yes	4 (80.0)	1 (20.0)			
Use of cocoa butter					
Yes	3 (50.0)	3 (50.0)	4.41	1.86-10.44	0.025^†^
No	33 (11.3)	258 (88.7)			
Any habits					
Yes	12 (11.4)	93 (88.6)	0.93	0.48-1.78	0.823
No	24 (12.3)	171 (87.7)			
Smoking					
Yes	3 (9.6)	28 (90.3)	0.79	0.26-2.42	1.000
No	33 (12.2)	236 (87.7)			
Alcohol drinking					
Yes	6 (9.5)	57 (90.4)	0.75	0.33-1.73	0.496
No	30 (12.6)	207 (87.3)			
Smoking and alcohol drinking					
Yes	3 (27.2)	8 (72.7)	2.39	0.86-6.60	0.133
No	33 (11.4)	256 (88.5)			
Number of cigarettes/day					
≥21	1 (20.0)	4 (80.0)	1.00		0.108
11-20	4 (30.8)	9 (69.2)	0.65	0.09-4.49	
1-10	1 (4.2)	23 (95.8)	4.48	0.35-64.56	
Alcoholic beverage consumption					
>2 doses/day	1 (4.8)	20 (95.2)	0.31	0.04-2.32	0.447
1 dose in last 30 days	8 (15.4)	44 (84.6)			

^#^ Pearson’s χ^2^ test; * R$= Reals. R$ 1.045,00 corresponds to US$ 193.96; ^†^ Statistically significant p values of 0.05 or less are highlighted in bold.

PR: prevalence ratio; 95%CI: 95% confidence interval; SPF: solar protection factor.

Regarding the risk factors associated with the development of AC, the results of bivariate analysis are shown in table 2 . There was a trend towards an association with cumulative sun exposure in years (p=0.067) and weekly sun exposure (p=0.059). The presence of AC was significantly associated with skin color (p<0.001) and daily sun exposure (p=0.05).

**Table 2:** Association between the presence of actinic cheilitis and risk factors in rural workers

Variables	Presence of actinic cheilitis		PR	95%CI	p value^**#**^
	Yes (%)	No (%)			
Cumulative sun exposure					
48-75 years	11 (11.5)	85 (88.5)	1.00		0.067
40-47 years	18 (17.5)	85 (82.5)	0.66	0.33-1.32	
14-39 years	7 (6.9)	94 (93.1)	1.65	0.67-4.09	
Skin color					
White	25 (23.4)	82 (76.6)	1.00		<0.001*
Brown	11 (6.2)	167 (93.8)	3.78	1.94-7.37	
Black	0 (0.0)	15 (100.0)	0.77	0.69-0.85	
Working hours					
Two shifts	33 (13.2)	217 (86.8)	2.20	0.70-6.89	0.153
One shift	3 (6.0)	47 (94.0)			
Daily sun exposure					
>9 hours	16 (17.6)	75 (82.4)	1.84	1.00-3.38	0.050*
Up to 9 hours	20 (9.6)	189 (90.4)			
Weekly sun exposure					
7 days	32 (14.0)	197 (86.0)	2.48	0.91-6.77	0.059
<7 days	4 (5.6)	67 (94.4)			

^#^ Pearson’s χ^2^ test; * Statistically significant p values of 0.05 or less are highlighted in bold.

PR: prevalence ratio; 95%CI: 95% confidence interval.

The multivariate model ( Table 3 ) identified a statistically significant association of the presence of AC with white ethnicity (p=0.001), male sex (p=0.002), duration of sun exposure >40 years (p=0.032), and elementary education *(* p=0.022). Thus, skin color, sex, and educational level of the patient, as well as cumulative sun exposure (in years), were identified as independent predictors of AC development.

**Table 3:** Multivariate analysis of the association between risk of actinic cheilitis and sociodemographic and occupational variables in rural workers

Variable	Reference	Predictor	PR	aPR	aCI*	p value
Skin color	White	Brown	0.30	0.32	0.16-0.63	0.001^†^
Sex	Female	Male	3.64	3.99	1.61-9.91	0.003^†^
Cumulative sun exposure (years)	14-39	40-47	1.92	2.04	1.06-3.92	0.032^†^
Educational level	Illiterate	Elementary school	1.97	2.45	1.14-5.28	0.022^†^

* aCI: confidence interval for prevalence ratio adjusted to a 5% level of significance (multiple analysis); ^†^ Statistically significant p values of 0.05 or less are highlighted in bold.

PR: prevalence ratio; aPR: adjusted prevalence ratio (logistic regression).

## DISCUSSION

Family agriculture currently accounts for 30% of Brazilian gross domestic product. In addition, this activity corresponds to 40% of Brazil’s active economy, according to the Brazilian Agricultural Research Corporation (EMBRAPA). According to data from the 2017 Agricultural Census, the number of farmers in Brazil was 10 million, 45% of them in the Northeastern region of the country. Compared to the general population, farmers are at higher risk of skin and lip cancer due to chronic exposure to UV radiation, no use of photoprotective measures, and tendency to have late diagnosis of AC.^( 2 , 13 )^

In the present research, there was a predominance of male, brown or white patients with a low educational level and low socioeconomic status. Similar results were reported by Miranda et al.,^( 9 )^ who analyzed the prevalence of AC lesions in 1,539 individuals from a rural population, who were exposed to sun during their work at a sugarcane plant. These findings can be explained by the fact that agriculture is a traditionally male activity in Brazil, because of the resistance and physical vigor of men, factors that facilitate performing the work.^( 13 )^

Studies investigating factors that impact the quality of life and health of people have shown that poor socioeconomic conditions directly influence not only the quality of life but also self-care, harmful occupational habits, and the level of knowledge about oral diseases.^( 14 - 16 )^ The present findings showed that the participants had a low income and low educational level. Additionally, most respondents seldomly sought dental care, although this service is offered by the rural worker’s union. These results are corroborated by the study of Leão et al.,^( 16 )^ who concluded that the higher the educational level, the more frequent seek for health services, including dental treatment.

Knowledge about AC is important for an early diagnosis, however few rural workers reported being aware of this potentially malignant lesion. Similar results were reported by Santos et al.,^( 11 )^ who found a low level of knowledge about AC among miners from the state of Paraíba, PB, Brazil. The prevalence of AC observed in the present study is in agreement with the rates described by de Souza Lucena et al.,^( 14 )^ de Oliveira Ribeiro et al.^( 17 )^ and Orozco et al.^( 18 )^ However, in a study conducted in 25 towns in the Seridó region, RN, Brazil, Ferreira et al.^( 13 )^ observed a higher prevalence of AC (28.4%), although the sample of the study was relatively larger (n=1,385).

In the present study, a marked predominance of AC lesions was found in male patients. This finding might be explained by the fact that women working in agricultural activities dedicate more time to domestic tasks, while men have a longer workday, are therefore exposed to sun for a longer period, and suffer greater consequences from the harmful effects of solar radiation. This explanation is consistent with previous studies that demonstrated a relation between cumulative sun exposure and development of AC.^( 9 , 11 , 13 , 19 )^ These results suggest the need to implement actions in primary care that encourage self-care by the male population guided by public policies, such as the National Policy of Comprehensive Men Health Care (PNAISH).^( 20 )^

With respect to the predominance of AC cases in the lower lip observed in the present study, Mello et al.^( 3 )^ emphasized this anatomic site is more exposed to solar radiation and is affected in 83.3% to 100% of cases. Our results also agree with those described by Silva et al.,^( 2 )^ who evaluated AC lesions diagnosed between 1953 and 2018, at 10 Brazilian Oral and Maxillofacial Pathology centers, and identified predominant involvement of the lower lip (97.3%). Curiously, Rodríguez-Blanco et al.^( 5 )^ found AC lesions in the upper lip, an uncommon finding in the literature.

Evaluation of the severity of AC showed a predominance of mild lesions in the two clinical presentations (acute and chronic). On the other hand, Miranda et al.^( 9 )^ found a higher frequency of moderate AC. However, to our knowledge, few studies in the English literature have compared acute and chronic lesions.

Some authors^( 17 , 19 )^ evaluated the clinical characteristics of AC, but did not associate them with the duration of cumulative sun exposure. On the other hand, Santos et al.^( 11 )^ concluded a work period of 14 years (cumulative sun exposure) is associated with the development of AC lesions. In the present study, individuals with 40 to 47 years of cumulative sun exposure were more frequently affected by AC. Taken together, the results suggest the longer the period of sun exposure, the higher the prevalence of AC.

All individuals diagnosed with AC reported the use of some photoprotective measure. The use of a hat was the most frequently cited measure, in agreement with the studies of Lucena et al.,^( 1 )^ Santos et al.,^( 11 )^ and Ferreira et al.^( 13 )^ Nonetheless, the present results provide evidence that the use of a hat alone does not effectively protect against the effects of solar radiation, since a significant association was found between the non-use of lip sunscreen and the presence of AC. The use of cocoa butter was frequent in the sample studied. In general, patients reported having started to use the product before the development of AC. However, the use of this product was discontinued after the appearance of signs and symptoms of AC. Within this context, in our sample, there was a significant association between AC and the use of cocoa butter, a lip balm that does not contain SPF. One hypothesis to explain this relation is the lack of knowledge about the effects of solar radiation. Instead of functioning as a protective barrier, cocoa butter applied to the lips facilitates the penetration of UV radiation, and thus contributes to the development of AC. Therefore, knowledge about effective photoprotective measures is important for the prevention of AC.

Previous studies^( 2 , 3 , 6 , 14 )^ have shown that fair-skinned people are more vulnerable to the harmful effects of UV radiation, because of the low level of melanin production, favoring the development of lesions caused by sun exposure, such as AC. Likewise, a higher prevalence of AC was observed among white compared to black participants.

Although the prevalence of AC was higher among subjects with elementary education, most of the participants in this study had a low educational level. This fact is consistent with the lack of knowledge about the importance of using photoprotection and adopting healthy lifestyle habits, and the failure to seek preventive health services, such as dental treatment, factors that contribute to the late diagnosis of lesions.

In contrast to other studies,^( 8 , 18 , 19 , 21 , 22 )^ the present results did not indicate a significant association between smoking and/or alcohol drinking, and the presence of AC. This finding can be explained by the small number of reports on the presence of these habits. However, Rodríguez-Blanco et al.,^( 5 )^ Santos et al.,^( 11 )^ and de Souza Lucena et al.,^( 14 )^ also found no significant relation between AC and these habits, although scientific evidence indicates these factors can increase the potential of malignant transformation of the disease.^( 3 , 8 )^ The present results suggest alcohol and cigarette consumption are not the main risk factors for the development of AC.

The limitations of this work were essentially the sample size for AC cases. However, the social relevance of the study lies in providing information to the study participants regarding AC and its risk of malignant transformation in lip squamous cell carcinoma. Within this context, our research contributed to instruct a vulnerable population about the necessary care to prevent AC and, consequently, lip cancer. Furthermore, the results were provided to the Rural Workers Union, responsible for offering dental services to research participants. The Union invited researchers to plan health promotion strategies through educational campaigns to prevent AC. This study may contribute to raise hypotheses that can be elucidated in subsequent research on the topic, such as longitudinal studies.

## CONCLUSION

Taken together, the results of this inquiry identified variables (skin color, sex, educational level, and duration of sun exposure) with significant impacts on the prevalence of actinic cheilitis. The most common clinical presentation was mild chronic actinic cheilitis. These findings highlight the need for educational and health intervention strategies designed for the population studied, to alert them about the etiology of actinic cheilitis and effective measures to prevent the disease, such as the use of lip balm with sun protection factor, sunscreen, and a wide brim hat. This study provided updated data on risk factors for the development of actinic cheilitis that contribute to the early diagnosis of this condition, and appropriate management of rural workers and other groups that perform outdoor occupations.
